# Influence of Maternal and Postweaning Linseed Dietary Supplementation on Growth Rate, Lipid Profile, and Meat Quality Traits of Light Sarda Lambs

**DOI:** 10.1155/2016/5391682

**Published:** 2016-03-13

**Authors:** Massimo Trabalza-Marinucci, Laura Mughetti, David Ranucci, Gabriele Acuti, Oliviero Olivieri, Dino Miraglia, Raffaella Branciari

**Affiliations:** Department of Veterinary Medicine, University of Perugia, Via San Costanzo 4, 06126 Perugia, Italy

## Abstract

The effects of dietary extruded linseed (EL) on growth performance, meat quality, and lipid profile of* Semimembranosus* and* Longissimus lumborum* muscles of 81 Sarda lambs were studied in a 3 × 3 design: EL content (0%, 10%, and 20%) of maternal dietary concentrate fed from 20 d to parturition to 60 d of lactation and EL content (0%, 10%, 20%) of lamb concentrate fed after weaning for 30 d. The basal diet was composed of alfalfa and meadow hay during pregnancy and alfalfa hay during lactation. At slaughter, carcass and meat quality were evaluated. Sensory quality of* Semimembranosus* from 0% and 20% EL lambs was assessed. Both maternal and postweaning diets affected growth performance, with higher body weights recorded with the 10% EL concentrate. Carcass and meat quality were not affected by diet. Saturated and monounsaturated FA decreased and n-3 polyunsaturated FA increased with increasing EL content in lamb diet. An increase in vaccenic and rumenic acid was associated with the EL content of the maternal diet. Both diets increased the n-6/n-3 FA ratio. No differences in acceptability were detected by consumers among groups. It is concluded that EL supplementation and early life nutrition can influence performance and FA metabolism in growing lambs.

## 1. Introduction

Ruminant meats have been associated with an increase in the risk of cardiovascular diseases, due to their high content of saturated fatty acids (SFA) [1, 2]. However, ruminant meats may also be a good dietary source of some nutrients with health benefits including a number of fatty acids (FA) such as long chain-polyunsaturated FA (LC-PUFA), n-3 PUFA in particular, and conjugated linoleic acid (CLA) isomers. The decrease of SFA and the increase of health-beneficial FA have been a main topic of ruminant meat research. Strategies for enrichment of lamb meat in n-3 PUFA have included diets enriched with fish products or with oilseeds such as linseed [3, 4]. However, many of the studies have focused on the effects of diets fed to growing animals [5–8]. Only a few studies, mainly addressed to laboratory and monogastric species [9–12], have investigated the effects of maternal consumption of LC-PUFA enriched diets during gestation and lactation on later performance and metabolism in the offspring. A correct status of LC-PUFA and a high ratio of n-3/n-6 PUFA are believed to be beneficial for growing animals that are facing the weaning and the derived stress. Since the quality of dietary fat can modify the cellular and lipid metabolism, it is expected that a maternal diet rich in n-3 PUFA can influence the lipid metabolism of the offspring in later life [10, 12–14]. However, this mechanism has not been fully explored. Hoile et al. [11] have demonstrated that maternal fat intake can alter the epigenetic regulation of* Fads2* transcription in rats. In goats, high *α*-linolenic acid diets were able to upregulate the peroxisome proliferator-activated receptors (PPAR-*α*) gene and downregulate the stearoyl-CoA-desaturase gene compared to control diets with low ALA content [15]. In a study conducted with Vendeen sheep, extruded linseed- (EL-) enriched concentrates used in both maternal and lamb weaning diets were able to increase the proportions of *α*-linolenic acid (ALA) and n-3 PUFA in tissues [16]. However, this experiment started three weeks after lambing and the lambs were assigned to the experimental diets when they were still suckling from their mothers.

In the Italian rearing systems, lambs are either fed maternal milk alone and slaughtered at 30–45 d of age or reared on a forage and concentrate diet and slaughtered before 4 months of age. Sarda is the most common dairy sheep breed found in Italy. In this work we investigated the effects of the interaction between maternal and postweaning diets, both enriched with EL, on growth rate, lipid profile, and sensory characteristics of meat from light Sarda lambs.

## 2. Materials and Methods

### 2.1. Experimental Design, Animals, and Diets

The present study was carried out in the “Azienda Zootecnica Didattica” of the Department of Veterinary Medicine, University of Perugia. Three weeks before their expected date of parturition, 60 Sarda ewes were randomly divided into three groups of equal size, balanced for body weight (BW: 45.5 ± 1.2 kg) and body condition score (2.9 ± 0.1) [17], and fed isoenergetic and isonitrogenous concentrates. The three concentrates were as follows: a control concentrate (CTR), without EL, and two experimental concentrates which contained, respectively, 100 g/kg (EL-10) and 200 g/kg (EL-20) of ground EL. Experimental diets were administered up to 60 days postpartum, when the lambs were weaned. Animals were fed 400 to 800 g per head per day of concentrate (during late pregnancy and early lactation, resp.), which was administered in two equal portions during the day. All the offered concentrate was consumed by the sheep during the whole length of the experiment. The concentrate composition is presented in Table 1. Alfalfa (crude protein: 12.4%; neutral detergent fiber: 44.5%; ash: 7.5%) and meadow (crude protein: 9.1%; neutral detergent fiber: 56.2%; ash: 7.3%) hay were provided in box feeders ad libitum. Average hay dry matter intake (measured over a period of 3 d/week as the difference between forage distributed and refusal) was 1.25 kg/ewe/day during pregnancy and 1.53 kg/ewe/day during lactation and was not affected by the dietary treatment.

Twinning rate was 38.3%. Lambs were suckled by their mothers up to 8 weeks of age and were housed in a stable with unlimited access to an outdoor paddock with no pasture available. At weaning (initial BW: 15.3 ± 0.8 kg) 81 lambs were randomly allocated to three groups of 27 animals each according to the EL content in the concentrate. The groups were balanced for sex, age, mothers' dietary treatment, and ram used in the breeding group. Lamb experimental concentrates were the same as the ones used in the ewes' diets (CTR, EL-10, and EL-20) and were offered daily at a rate of 50 and 150 g/head during the first 10 d and the remaining 20 d of the trial, respectively. The concentrate was entirely consumed by the animals. Lambs were offered the ewes' alfalfa hay ad libitum. The estimated hay dry matter intake averaged 0.55, 0.53, and 0.52 kg/lamb/day for CTR, EL-10, and EL-20 groups, respectively.

At the end of trial, lambs were transported to the abattoir. After weighing, to obtain the live slaughter weight, lambs were stunned and slaughtered by exsanguination.

### 2.2. Recordings, Sampling, and Analytical Procedures

During the trial, samples of experimental concentrates were collected weekly and analysed for chemical composition following AOAC methods [18–21]. The methods of Van Soest et al. [22] were used in the analyses of NDF (not assayed with a heat stable amylase), ADF, and lignin (sa). Sodium sulphite was used in the NDF procedure, and both NDF and ADF were expressed inclusive of ash.

Lambs were weighed at birth, at weaning, and at slaughter. Carcasses were weighed after 24 hours of chilling, before dissection, to obtain the cold carcass weight. Ten carcasses of each group were randomly selected for further analyses.

The pH was assessed 15 minutes after slaughter on* Longissimus lumborum* (LL) muscle (space between last thoracic and first lumbar vertebrae) and on* Semimembranosus* (SM) muscle using a puncture electrode probe connected to a portable pH meter (model MP120, Mettler Toledo Inc., Columbus, USA). The carcasses were then chilled at a temperature of 5°C. The entire LL (between the 2nd-3rd last thoracic vertebrae and the 4th-5th lumbar vertebrae) and SM muscles from the right side of the carcasses were removed 24 h postmortem and representative subsamples of the muscles were obtained to assess ultimate pH, color, cooking loss, Warner-Bratzler shear (WBS) force, proximate composition, cholesterol content, and FA profile.

Meat color was estimated in 6 different surface points of a 2.5 cm thick sample of both muscles (MLS on the 12th rib) subjected to a 1 h blooming period at 4°C after cutting using a tristimulus colorimeter (CR-400 Chroma Meter, Minolta Ltd., Osaka, Japan) and expressed as CIE LAB *L* ^*∗*^ *a* ^*∗*^ *b* ^*∗*^ values [23]. For the cooking loss determination, LL and SM samples (1.5 cm thick) were weighed and placed in thin-walled plastic bags in a water-bath at 80°C. After 1 h the samples were removed from the water-bath, cooled in cold water, dry blotted, and weighed. Cooking loss was calculated as the difference in sample weight before and after cooking, expressed as a percentage of the initial sample weight [24]. The WBS measurements of the cooked LL and SM samples (same samples as used for cooking loss determination) were obtained with a WBS device fitted to an Instron Universal Testing Machine (Model 1011) with a 50 kg load cell (Instron, Norwood, MA, USA). These readings were taken within 1 h of the final weight measurement (cooking loss determination). Three cylindrical cores were cut from each muscle using a 1,2265 cm diameter core. Samples were randomly removed from the center of each muscle. The maximum WBS values (kg/cm^2^) required to shear a cylindrical core of cooked muscle, perpendicular to the muscle fiber longitudinal axis (at a crosshead speed of 100.0 mm/min), were recorded for each core in triplicate and the mean was calculated for each muscle [24].

Proximate composition of LL and SM muscles samples was analysed according to AOAC [21]. Total cholesterol of the muscles was determined by colorimetric kit (Cat. number 10139050035: Cholesterol Biopharm, Germany) with a spectrophotometer (Ultrospec 2011 pro, Amersham Pharmacia Biotech, Milan, Italy) at a wavelength of 405.

Milk FA were extracted according to the Röse-Gottlieb method [18], while meat FA were extracted according to the Bligh and Dyer [25] method. The transmethylation was performed according to the procedure of Branciari et al. [26]. Methyl esters were separated and quantified using a VARIAN 3400 gas chromatograph equipped with a flame ionization detector (FID) and a split-splitless injector. Analyses were performed with CP-Select CB for FAME fused silica capillary column (100 m × 0.25 mm i.d., film thickness of 0.39 *μ*m, J&W, Agilent technologies, Palo Alto, CA, US). The injection volume was 1 *μ*L. The carrier gas was high purity helium with flow rate of 1 mL/min. The injector and detector temperatures were kept at 290°C. The column oven temperature was programmed at 120°C and increased by 3.2°C/min up to 170°C and then increased by 2.1°C/min from 170°C to 225°C. Individual FA methyl esters were identified by comparison with a standard mixture containing 37 FAMEs (Supelco, Bellefonte, PA, USA) and other polyunsaturated FAMEs, as cis-11-vaccenic methyl ester, trans-11-vaccenic methyl ester, and methyl cis-7,10,13,16,19-docosapentaenoate (Sigma-Aldrich, Bellefonte, PA, USA). Quantification was carried out using nonadecanoic acid as internal standard (C19:0, Supelco Park, Bellefonte, PA, USA) added to the sample at the time of extraction.

### 2.3. Sensory Evaluation of Lamb Thigh

Staff and students of the Veterinary Medicine Department of the University of Perugia were recruited as members of a consumer panel. Consumers were asked to complete a questionnaire that included information about age, sex, and the frequency of consumption of lamb meat. In total 100 consumers, aged 18 to 65, participated in the test. The evaluation was conducted in two different sessions with 60 and 40 consumers in the 1st and 2nd sessions, respectively, and using meat patty samples obtained from the SM muscle after approximately 1 month of frozen storage. Vacuum-packed thighs (2 kg) were thawed for 36 h at +4°C. Patties were produced from boneless thigh meat obtained from CTR and EL-20 lambs. Thighs were pooled within each group, ground, and formed into 4 cm diameter patties in a patty machine. Thigh patties from both CTR and EL-20 groups were divided into two subgroups and cooked either without any seasoning or with salt, spices, and olive oil. Patties were placed on steel trays covered with aluminum foil and oven cooked at 170°C (10% relative humidity) for approximately 2 h. The patties were kept warm until served. Each participant evaluated 4 different patties (CTR and EL-20, both with or without seasoning).

A practicing session was performed before the test to allow consumers to become familiar with the use of the scale and explain the definitions of attributes. Samples were monadically served on white plastic plates identified by three random digit codes. Consumers received no information and were asked to rate sensory attributes (lamb flavour, lamb odour, extraneous flavours, extraneous odours, and overall acceptability) using an unstructured scale (0–9) [27].

### 2.4. Statistical Analyses

All data were analysed using an ANOVA in the GLM procedure of SAS [28]. The statistical model included maternal diet (CTR, EL-10, and EL-20), lamb diet (CTR, EL-10, and EL-20), muscle type (LL and SM), and their interactions. For the sensory data, the model used included lamb diet (CTR, EL-10, and EL-20), presence of seasoning, and age, gender, and geographical origin of the assessor. Frequency of lamb meat consumption was included as a covariate. The effect of maternal diet was found to be not significant (*P* > 0.05) and was removed from the model. Overall differences between the means were evaluated using Tukey's test. Data were reported as least squares means ± standard error. Differences were considered to be significant when *P* ≤ 0.05.

### 2.5. Ethical Rules

Use and care of animals in this study were in accordance with the European recommendations [29] for the protection of animals used for scientific purposes.

## 3. Results and Discussion

### 3.1. Milk Composition

Ewe's milk composition was not affected by the dietary treatment and had the following average characteristics: pH 6.67 ± 0.03; 4.69 ± 0.09% w/w lactose; 6.55 ± 0.10% w/w fat; and 6.01 ± 0.12% w/w protein (MilkoScan 6000, Foss Electric, Hillerød, Denmark).

EL supplementation reduced the proportions of SFA from C6:0 to C17:0 (Table 2). The level of most 18-carbon FAs (C18:0, C18:1, C18:2, C18:3, and isomers) was influenced by the dietary treatment: in particular, the vaccenic acid (C18:1 t11) percentage increased more than 3-fold with the EL-20 diet. Stearic acid (C18:0) and oleic acid (C18:1 c9) were also positively influenced by the dietary treatment. A significant increase of some isomers of C18:2 was also observed following EL dietary supplementation. The concentration of rumenic acid (C18:2 c9 t11) showed a trend similar to that of vaccenic acid. An increase in the percentage of ALA values was also found. The arachidonic acid (C20:4 n-6) was the PUFA present in greatest concentration in the CTR milk and decreased with EL supplementation. LC-PUFA n-3 (C20:5, C22:6) percentage in milk was not modified by diet and was below 0.2%. These results showed on the whole that the EL supplementation decreased the percentage of SFA and increased the MUFA and PUFA contents, thus contributing to modifying the P/S ratio and the n-6/n-3 ratio. These results are in agreement with most data published in the literature [26, 30, 31].

### 3.2. Growth Performance

The BW at birth and growth performance of lambs before weaning were not affected by the dietary treatment of the mothers (Table 3). These data are in agreement with Gallardo et al. [32], who examined the performance of Churra lambs slaughtered at a BW of 12 kg and fed with the milk of ewes receiving linseed oil-enriched (2.6%) diets. In this experiment, no dietary effects on growth performance were observed.

In contrast, the postweaning growth rate was influenced by both lamb diet and maternal feeding regimen, with the EL-10 treatment being the most effective in improving the performance (Table 3). The intermediate level of dietary EL (and the limited daily intake of concentrate) was possibly able to provide the rumen environment with the optimal amount of lipids and, in the same time, did not impair dry matter degradability. It has been shown that at the highest EL concentration used in this study (20% of the concentrate) ruminal degradability and milk production can be negatively affected in dairy ewes [33, 34]. Berthelot et al. [16] evaluated the effects of EL-enriched diets administered to either Vendeen ewes or their lambs in 2 × 2 factorial arrangement using lower EL contents in both maternal (12.5%) and lamb (6%) concentrates compared to the EL levels used in the present study. In their experiment, no dietary effects were observed on lamb growth performance before and after weaning. When analysing the effects of diets which differed in their n-6/n-3 FA ratio (from 2.3 to 15.6) and included linseed oil, soybean oil, or cottonseed oil, Kim et al. [35] did not report differences in BW variations among experimental groups. In contrast, Ponnampalam et al. [8] examined the effects of different forms of dietary n-3 FA on ewe lambs performance and found that whole linseed added to the diet (10%) improved carcass yield, feed : weight gain, and final BW. Jerónimo et al. [36] replaced sunflower oil with linseed oil in Merino Branco ram lambs' (average initial BW: 23 kg) diets and found a linear increase in live slaughter weight and hot carcass weight. It must be stressed, however, that comparisons should be interpreted with caution since most experimental factors (genotype, diet composition, feed intake, age at the beginning of the trial, average daily gain, and BW at slaughter) markedly differ between the published studies and the present work.

As for the maternal diet effect, a possible epigenetic influence can be hypothesized. There are no experimental data which support this hypothesis in sheep; however, a maternal high-fat diet was shown to increase offspring BW in mice [9]. These BW changes did not correlate with feed or energy intake, but they were rather associated with an epigenetic influence of the GH/IGF-I axis. Indeed, the nutritional status experienced by animals around birth has implications not only for later life risks of developing diseases but also for adipose tissue growth and serum hormone levels [37]. In a series of animal studies, offspring of mothers fed a diet high in calories or high in fat before birth were heavier and had a higher percentage of body fat throughout life [10]. From an evolutionary point of view, the modification of the body size would be a proper response to changing factors such as food availability [38]. In addition, it has been demonstrated in pigs that a maternal diet rich in linseed oil during gestation and lactation can significantly modify the FA composition, structure, and physiology of the offspring ileum, thereby influencing the digestive processes [14].

### 3.3. Meat Quality Traits

The meat quality results are reported in Table 4. No differences were recorded between the LL and SM muscles for all the parameters examined in this study (data not shown). For this reason, data reported in Tables 4 and 5 were pooled across muscles.

The lamb diet was able to affect meat pH and lightness values recorded after 24 hours: EL-10 samples showed a slight but significant lower pH than the EL-20 ones and a higher *L* ^*∗*^ value when compared to CTR. Neither lamb nor maternal diet was able to affect proximal chemical composition and cholesterol content of the meat. These results show that lamb meat has similar chemical traits regardless of the EL content of both maternal and lamb diets. The differences recorded for the pH and color coordinate values, even if statistically significant, are of limited value. Furthermore, no effects on the other parameters generally linked with muscle pH and lightness, that is, cooking loss, were observed. These results are in accordance with Moloney et al. [39], who reported similar trends in final pH and color of SM and LL of lambs fed with linseed oil and NaOH-treated linseed compared to a control group. Similarly, only few differences were reported in the meat composition of Manchego lambs fed with EL, when compared to control animals, and no effects were recorded in the meat of lambs derived from Churra ewes fed with a linseed oil-enriched diet [7, 40]. However, the comparison with data reported by other authors should be carefully considered as the differences between experimental factors can influence the final meat quality characteristics.

The fatty acid composition of the meat was markedly modified by the lamb dietary treatment (Table 5). The proportion of SFA from 12:0 to 17:0 decreased with the EL dietary supplementation. The EL supplementation modified the level of most 18-carbons and increased the stearic acid concentration. A remarkable increase of vaccenic acid, linoleic acid (C18:2 c9 c12), ALA, and the sum of LC-PUFA n-3 was induced by the EL-20-enriched diet. These results are in agreement with data obtained by de la Fuente-Vázquez et al. [7] with Manchego lambs fed a concentrate containing 8.7% EL. In this study a decrease in SFA and MUFA and an increase in PUFA and n-3 FA, C18:3 n-3 in particular (6.5-fold higher compared to control), was observed. The C18:3 n-3 increase is strongly related to the presence of linseed in the diet, with this oilseed being the major dietary source of ALA [41, 42]. As for the long chain n-3 FA, they are recovered only in limited amounts in meat as herbivore diets normally lack these FA (unless marine sources are used). However, results from the present study reinforce the hypothesis that a conversion of ALA to LC-PUFA n-3 through elongation and desaturation in tissues is possible [16, 36]. The competition between C18:2 n-6 and C18:3 n-3 for desaturation enzymes might affect the conversion to long chain derivatives. The higher proportion of LC-PUFA n-3 in total n-3 FA, compared to the proportion of LC-PUFA n-6 in total n-6 FA, is likely due to the preference of these enzymes for ALA [36].

Table 5 shows the influence of the maternal diet during gestation and lactation on the FA composition of lamb meat. Major effects can be found on SFA (C12:0 and C14:0), MUFA (C18:1 t11), and PUFA, among which rumenic acid (C18:2 c9 t11) and DHA (C22:6 n-3) increased and C20:4 n-6 decreased. To our knowledge, there are no published data concerning the effects of the nutritional status experienced by lambs around birth on the FA metabolism in later life. It has been demonstrated in rats that type of fat fed to mice during gestation and lactation can affect the influence of the dietary fat after weaning on the FA composition of liver and hearth in the offspring [43]. Hoile et al. [11] showed that maternal fat intake in rats was able to modify, through epigenetic regulation, the messenger RNA expression of the genes encoding Δ-5 and Δ-6 desaturases (*Fads1* and* Fads2*) and alter the C20:4 n-6 and C22:6 n-3 status in liver. Even if these results cannot be entirely transposed to the ovine species, it can be hypothesized that a similar epigenetic regulation of the FA synthesis exists. In a recent study with goats fed with a linseed oil-supplemented diet, Ebrahimi et al. [15, 44] found an effect on the expression of the PPAR-*α* gene associated with the regulation of FA metabolic pathway and a downregulation of the SCD gene expression.

### 3.4. Sensory Analysis

The consumer analysis did not reveal any effect on overall acceptability and sensory properties of meat, except for a higher perception of extraneous flavours in the EL-20 samples (Table 6). This effect was influenced by the presence of seasoning (salt, olive oil, and spices) as well, being samples without seasoning characterised by a higher score for extraneous flavours (2,3 versus 0.2 for samples with or without seasoning, resp.) (Table 6). There are not many reports in the literature showing the effects of dietary linseed on the sensory quality of the cooked lamb meat. As reported by several authors [45, 46], the use of dietary n-3 PUFA can affect the susceptibility to oxidation of the meat and induce flavours defects. After feeding lambs protected PUFA from sunflower seeds, higher notes for extraneous aroma and flavour and decreased lamb meat typical flavour were reported by Park et al. [47]. In another study, where steers were fed diets containing the same linseed concentration as that used in the present experiment (0.2% of BW), a perception of extraneous flavours was recorded [48]. In contrast, lamb steaks obtained from animals that received linseed oil-enriched diet were given the highest scores for lamb flavour and overall liking and the lowest score for “abnormal” lamb flavour [49].

Finally, the sensory properties of meat were affected to variable extent by age, gender, and geographical origin of the assessor (data not shown).

## 4. Conclusions

Nutritionists recommend an increase in the intake of LC-PUFA n-3 and CLA, as well as an increase in the ratio of PUFA to SFA and/or in the ALA to C18:2 n-6 ratio in dietary lipids. Results from the present experiment confirm that dietary linseed can effectively modify the lipid composition of lamb meat and increase the amounts of FA with nutraceutical activity.

In addition, the level of EL supplementation in both maternal and lamb feeds appeared to positively affect growth performance, while it did not significantly influence the sensory properties of the meat.

Although the data obtained need further confirmation by molecular biology studies in the ovine species, it is suggested that the nutritional environment in early life provides cues for fetal development and can be crucial for FA metabolism throughout life.

## Figures and Tables

**Table 1:** Ingredients and chemical composition of the experimental concentrates fed during the trial.

Item	Concentrates^1^		
	CTR	EL-10	EL-20
Raw materials (% as fed basis)			
Soybean meal	188.2	124.0	60.8
Corn meal	335.9	157.0	20.0
Barley grain	50.0	50.0	20.0
Wheat bran	162.6	303.1	578.2
Wheat flour shorts	200.0	200.0	57.4
Extruded linseed	—	100.0	200.0
Molasses	20.0	20.0	20.0
Calcium carbonate	16.9	19.7	21.6
Bicalcic phosphate	9.4	4.2	—
Sodium bicarbonate	5.0	5.0	5.0
Sodium chloride	5.0	5.0	5.0
Vitamin and mineral premix^2^	5.0	5.0	5.0
Bonding agent	—	5.0	5.0
Magnesium oxide	2.0	2.0	2.0

Analysed nutrients^3^ (g/100 g)			
DM	89.12	88.66	89.12
CP	15.99	15.85	16.01
CF	2.53	6.93	10.23
Ash	6.83	7.25	7.83
NDF	16.33	19.23	24.34
ADF	5.01	6.21	7.99
Lignin	1.12	1.46	2.31
Calcium	1.28	1.35	1.41
Phosphorus	0.35	0.40	0.41

C16:0	15.50	10.11	8.90
C18:0	2.09	2.97	3.42
C18:1 c9	22.78	18.11	17.59
C18:2 c9, c12	54.07	32.64	23.86
C18:3 c9, c12, c15	5.32	35.22	45.41

^1^CTR: diet without extruded linseed; EL-10: diet containing 100 g/kg of extruded linseed; EL-20: diet containing 200 g/kg of extruded linseed.

^2^Mineral and vitamin premix supplied (per kg of final diet): Co, 0.30 mg (2CoCO_3_; 3Co(OH)_2_ H_2_O); Zn, 50.00 mg (ZnO); Fe, 15.00 mg (FeCO_3_); Mn, 30.00 mg (MnO); Se, 0.60 mg (Na_2_SeO_3_); I, 1.00 mg (Ca(IO_3_)_2_); vitamin A, 50,000 IU (retinylacetate); cholecalciferol, 3,000 IU; vitamin E, 50.00 mg (*α*-tocopherol acetate).

^3^DM: dry matter; CP: crude protein; CF: crude fat; NDF: neutral detergent fibre; and ADF: acid detergent fibre.

**Table 2:** Effect of linseed supplementation on the fatty acid profile (g/100 g of total fatty acids methyl esters) of milk.

	Maternal diet			SEM	*P*
	CTR	EL-10	EL-20		
C4:0	3.38	3.61	3.39	0.081	0.433
C6:0	2.84^a^	2.51^b^	1.83^c^	0.108	<0.001
C8:0	2.97^a^	2.44^b^	1.53^c^	0.148	<0.001
C10:0	9.38^a^	7.06^b^	4.54^c^	0.494	<0.001
C11:0	0.46^a^	0.30^b^	0.25^b^	0.031	0.004
C12:0	5.23^a^	3.91^b^	2.51^c^	0.272	<0.001
C13:0	0.20^a^	0.15^b^	0.10^c^	0.010	<0.001
C14:0	11.46^a^	9.64^b^	8.04^c^	0.350	<0.001
C14:1	0.27^a^	0.17^b^	0.11^c^	0.016	<0.001
C15:0	1.18^a^	1.15^a^	1.03^b^	0.021	0.003
C16:0	26.38^a^	21.55^b^	18.95^c^	0.754	<0.001
C16:1	1.54^a^	1.16^b^	0.95^c^	0.060	<0.001
C17:0	0.82^a^	0.79^a^	0.70^b^	0.017	0.002
C17:1	0.25^a^	0.21^b^	0.15^c^	0.010	<0.001
C18:0	6.58^a^	9.38^b^	10.90^c^	0.450	<0.001
C18:1 *trans-9*	0.38^a^	0.67^b^	0.75^c^	0.039	<0.001
C18:1 *trans-11*	2.28^a^	4.27^b^	7.22^c^	0.439	<0.001
C18:1 *cis-9*	14.28^a^	17.55^b^	18.69^c^	0.462	<0.001
C18:2 *trans-9*, *trans-12*	0.04^a^	0.14^b^	0.35^c^	0.033	<0.001
C18:2 *cis-9*, *cis-12*	2.79	2.81	2.81	0.020	0.883
C18:2 *cis-9*, *trans-11*	0.72^a^	1.44^b^	2.18^c^	0.145	<0.001
C18:3 n-3	1.14^a^	1.53^b^	1.96^c^	0.082	<0.001
C18:3 n-6	0.04^a^	0.02^b^	0.01^b^	0.005	0.010
C20:0	0.28	0.28	0.27	0.004	0.694
C20:4 n-6	0.16^a^	0.07^b^	0.03^b^	0.014	0.002
C20:5 n-3	0.04	0.05	0.05	0.004	0.586
C21:0	0.07	0.06	0.05	0.009	0.636
C22:0	0.11	0.14	0.14	0.009	0.250
C22:5 n-3	0.11	0.12	0.12	0.004	0.559
C22:6 n-3	0.01	0.01	0.01	0.002	0.417
C23:0	0.07	0.06	0.06	0.010	0.984
C24:0	0.05	0.04	0.04	0.008	0.893
n-6/n-3	2.26^a^	1.72^b^	1.48^c^	0.084	<0.001
SFA	71.46^a^	63.07^b^	54.32^c^	1.707	<0.001
PUFA	5.11^a^	6.25^b^	7.61^c^	0.251	<0.001
MUFA	19.02^a^	24.03^b^	27.88^c^	0.885	<0.001
P/S	0.07^a^	0.10^b^	0.14^c^	0.007	<0.001

CTR: diet without extruded linseed; EL-10: diet containing 100 g/kg of extruded linseed; EL-20: diet containing 200 g/kg of extruded linseed; SFA, saturated fatty acids; MUFA, monounsaturated fatty acids; PUFA, polyunsaturated fatty acids; P/S, polyunsaturated/saturated fatty acids.

Means with different superscript within the same row differ.

**Table 3:** Effect of maternal and lamb postweaning diets on body weight (BW, kg), average daily gain (ADG, kg/die), and cold carcass weight of light Sarda lambs.

	Maternal diet (M)			SEM	Postweaning diet (PW)			SEM	*P*
	CTR	EL-10	EL-20		CTR	EL-10	EL-20		M × PW
BW at birth	3.48	3.65	3.49	0.09					
BW at weaning	16.64	15.41	15.89	0.65					
ADG at weaning	0.206	0.184	0.201	0.01					
BW at slaughter	18.46^b^	19.22^a^	18.83^ab^	0.32	18.97^AB^	19.32^A^	18.58^B^	0.29	0.281
ADG at slaughter	0.074^b^	0.103^a^	0.092^ab^	0.01	0.090^AB^	0.107^A^	0.073^B^	0.01	0.318
Cold carcass weight	8.96^b^	9.29^a^	9.04^ab^	0.35	9.13^ab^	9.34^a^	9.15^b^	0.22	0.565

CTR: diet without extruded linseed; EL-10: diet containing 100 g/kg of extruded linseed; EL-20: diet containing 200 g/kg of extruded linseed.

Means with different superscript within the same row differ. ^a,b^ *P* < 0.05; ^A,B^ *P* < 0.01.

**Table 4:** Effect of maternal and lamb postweaning diets on meat quality traits of light lamb meat.

Item	Maternal diet (M)			SEM	Postweaning diet (PW)			SEM	*P*
	CTR	EL-10	EL-20		CTR	EL-10	EL-20		M × PW
pH, 15 min	6.64	6.68	6.59	0.04	6.61	6.62	6.68	0.05	0.384
pH, 24 h	5.98	5.98	5.94	0.01	5.97^ab^	5.94^b^	6.00^a^	0.02	0.139

Raw meat colour									
*L* ^*∗*^	43.28	43.74	44.79	0.67	42.56^b^	45.44^a^	43.82^ab^	0.78	0.174
*a* ^*∗*^	23.02	22.90	22.99	0.46	23.15	22.14	23.61	0.54	0.243
*b* ^*∗*^	6.44	6.47	6.68	0.24	6.43	6.38	6.79	0.29	0.489

Cooking loss (%)	25.15	24.44	24.66	0.77	24.70	25.36	24.20	0.91	0.683
WB shear force (N/cm^2^)	5.12	5.70	5.36	0.14	4.83	5.53	5.82	0.17	0.925

Protein (%)	19.86	18.46	19.12	0.36	19.41	18.94	19.09	0.41	0.962
Lipid (%)	3.06	3.23	2.96	0.14	3.11	3.02	3.11	0.16	0.483
Ash (%)	1.03	1.02	1.03	0.03	1.01	1.04	1.03	0.02	0.219
Cholesterol (mg/100 g)	53.56	51.81	58.37	3.15	52.69	59.55	51.49	3.47	0.246

CTR: diet without extruded linseed; EL-10: diet containing 100 g/kg of extruded linseed; EL-20: diet containing 200 g/kg of extruded linseed.

Means with different superscript within the same row differ at *P* < 0.05.

**Table 5:** Effect of maternal or lamb postweaning diet on the fatty acid composition of light lamb meat (g/100 g of fatty acids methyl esters).

Item	Maternal diet (M)			SEM	Lamb postweaning diet (PW)			SEM	*P*
	CTR	EL-10	EL-20		CTR	EL-10	EL-20		M × PW
C10:0	0.109	0.137	0.108	0.021	0.145	0.115	0.095	0.026	0.181
C12:0	0.463^A^	0.333^B^	0.335^B^	0.021	0.435^A^	0.401^A^	0.296^B^	0.023	<0.001
C14:0	4.240^a^	3.449^b^	3.648^b^	0.012	4.296^A^	3.680^B^	3.360^B^	0.148	<0.001
C14:1	0.091	0.042	0.050	0.018	0.100	0.055	0.027	0.022	0.048
C15:0	0.559	0.554	0.515	0.023	0.535	0.543	0.549	0.028	0.188
C16:0	21.618	20.413	20.076	0.031	22.220^**A**^	20.446^**B**^	19.441^**B**^	0.375	0.068
C16:1	1.890^**A**^	1.313^**B**^	1.460^**AB**^	0.067	1.837^A^	1.454^B^	1.372^B^	0.082	0.127
C17:0	1.026	1.001	1.008	0.019	1.073^**A**^	1.040^**A**^	0.921^**B**^	0.025	0.769
C17:1	0.033	0.182	0.078	0.031	0.155	0.091	0.047	0.038	0.311
C18:0	12.708	13.950	13.478	0.262	12.531^b^	13.673^ab^	13.932^a^	0.321	0.284
C18:1 t n-9	0.161	0.073	0.124	0.043	0.076	0.206	0.077	0.055	0.256
C18:1 c n-9	26.703	26.160	27.854	0.512	28.701^A^	26.678^AB^	25.337^B^	0.653	0.379
C18:1 t 11	3.027^b^	3.716^ab^	4.040^a^	0.165	3.050^b^	3.810^ab^	3.921^a^	2.211	0.217
C18:2 t n-6	0.020^ab^	0.018^b^	0.061	0.011	0.034	0.009	0.055	0.014	0.199
C18:2 c n-6	10.613	11.115	10.221	0.399	9.210^B^	10.877^AB^	11.862^A^	0.510	0.053
C18:2 c9,t11	1.034^B^	1.238^AB^	1.456^A^	0.071	1.139	1.293	1.296	0.090	0.829
C18:3 n-3	2.422	2.759	2.669	0.087	2.041^**C**^	2.714^**B**^	3.096^**A**^	0.107	0.066
C20:0	tr.	tr.	0.003	0.003	0.003	tr.	tr.	0.003	0.883
C21:0	tr.	0.006	0.021	0.008	0.021	0.006	tr.	0.007	0.088
C22:0	tr.	tr.	0.021	0.011	0.009	0.012	tr.	0.014	0.916
C20:4 n-6	4.347^A^	3.774^AB^	3.333^B^	0.171	4.053	3.529	3.872	0.210	0.036
C20:3 n-6	0.189^b^	0.273^a^	0.222^ab^	0.015	0.188	0.233	0.263	0.019	0.107
C23:0	0.171^AB^	0.207^A^	0.121^B^	0.017	0.161	0.133	0.203	0.020	0.001
C20:5 n-3	0.793	0.963	0.945	0.071	0.718	0.957	1.026	0.087	0.539
C22:5 n-3	1.211	1.312	1.103	0.068	1.009^b^	1.270^ab^	1.346^a^	0.083	0.553
C22:6 n-3	0.307^b^	0.420^a^	0.354^ab^	0.019	0.324	0.362	0.395	0.023	0.008
SFA	40.893	40.051	39.333	0.446	41.429^a^	40.049^ab^	38.799^b^	0.546	0.035
MUFA	31.905	31.486	33.605	0.614	33.920^a^	32.294^ab^	30.782^b^	0.752	0.395
Other MUFA	2.014^a^	1.537^b^	1.588^b^	0.081	2.092^**A**^	1.600^**B**^	1.446^**B**^	0.111	0.205
PUFA	20.936	21.873	20.363	0.719	18.717^b^	21.244^ab^	23.212^a^	0.914	0.104
n-6	15.169	15.180	13.836	0.550	13.485	14.647	16.053	0.674	0.081
n-3	4.733	5.454	5.071	0.219	4.093^**B**^	5.304^**A**^	5.863^**A**^	0.268	0.246
n-3 LC-PUFA	2.311	2.695	2.403	0.145	2.052^b^	2.590^ab^	2.767^a^	0.178	0.391
n-6/n-3	3.419^**A**^	2.798^**B**^	2.759^**B**^	0.076	3.380^**A**^	2.813^**B**^	2.783^**B**^	0.097	0.026
IDCLA	26.591	25.140	26.268	0.570	27.927^A^	25.599^AB^	24.473^B^	0.698	0.212
ATH	0.763^a^	0.665^b^	0.669^b^	0.021	0.777^**A**^	0.687^**B**^	0.634^**B**^	0.022	0.001
THR	1.045	0.954	0.955	0.025	1.088^A^	0.969^B^	0.898^B^	0.031	0.054

CTR: diet without extruded linseed; EL-10: diet containing 100 g/kg of extruded linseed; EL-20: diet containing 200 g/kg of extruded linseed.

SFA: saturated fatty acids.

MUFA: monounsaturated fatty acids.

PUFA: polyunsaturated fatty acids.

n-3 LC-PUFA: n-3 long chain-polyunsaturated fatty acids.

IDCLA: index of the Δ9-desaturase activity = C18:2 cis 9, trans-11 × 100/C18:1 trans-11 + C18:2 cis-9, trans-11.

ATH: atherogenicity index = C12:0 + (4 × C14:0 + C16:0)/MUFA + PUFA.

THR: thrombogenicity index = C14:0 + C16:0 + C18:0/(0.5 × MUFA + 0.5 × n-6 PUFA + 3 × n-3 PUFA + n-3 PUFA/n-6 PUFA).

Tr: traces, under the detectable limits.

Means with different superscript within the same row differ. ^a,b^ *P* < 0.05; ^A, B^ *P* < 0.01; ^**A**, **B**, **C**^ *P* < 0.001.

**Table 6:** Effect of lamb postweaning (PW) diet and presence of seasoning (S) on sensory properties of light lamb meat.

Item	CTR		EL-20		SEM	*P*	
	With seasoning	Without seasoning	With seasoning	Without seasoning		PW	S
Lamb meat odour	5.357	5.740	5.373	5.917	0.520	0.762	0.387
Extraneous odour	1.342	2.364	1.748	3.021	0.601	0.152	0.065
Lamb meat flavour	5.162	4.967	5.578	5.788	0.568	0.079	0.990
Extraneous flavour	0.100	2.026	0.406	2.733	0.516	0.014	<0.001
Overall liking	7.089	5.899	6.606	5.055	0.678	0.114	0.051

CTR: diet without extruded linseed; EL-20: diet containing 200 g/kg of extruded linseed.

Seasoning: salt, olive oil, and spices.
